# Improved Deep Residual Shrinkage Network for Intelligent Interference Recognition with Unknown Interference

**DOI:** 10.3390/s23187909

**Published:** 2023-09-15

**Authors:** Xiaojun Wu, Yibo Zhou, Daolong Wu, Haitao Xiao, Yaya Lu, Hanbing Li

**Affiliations:** 1 School of Software Engineering, Xi’an Jiaotong University, Xi’an 710049, China; xiaojunw@xjtu.edu.cn (X.W.); zhouyibo@stu.xjtu.edu.cn (Y.Z.); lyy2083024832@stu.xjtu.edu.cn (Y.L.); 2 Shaanxi Joint Laboratory of Artificial Intelligence, Xi’an Jiaotong University, Xi’an 710049, China; 3 Key Laboratory of Technology on Datalink, China Electronics Technology Group Corporation (CETC), 20th Institute, Xi’an 710068, China; 4 School of Information and Communication Engineering, Xi’an Jiaotong University, Xi’an 710049, China; xht8015949@xjtu.edu.cn; 5 Songshan Laboratory, Zhengzhou 450046, China; anatoly-li@foxmail.com

**Keywords:** communication interference, soft threshold, SNCS, OCSVM, new class rejection

## Abstract

In complex battlefield environments, flying ad-hoc network (FANET) faces challenges in manually extracting communication interference signal features, a low recognition rate in strong noise environments, and an inability to recognize unknown interference types. To solve these problems, one simple non-local correction shrinkage (SNCS) module is constructed. The SNCS module modifies the soft threshold function in the traditional denoising method and embeds it into the neural network, so that the threshold can be adjusted adaptively. Local importance-based pooling (LIP) is introduced to enhance the useful features of interference signals and reduce noise in the downsampling process. Moreover, the joint loss function is constructed by combining the cross-entropy loss and center loss to jointly train the model. To distinguish unknown class interference signals, the acceptance factor is proposed. Meanwhile, the acceptance factor-based unknown class recognition simplified non-local residual shrinkage network (AFUCR-SNRSN) model with the capacity for both known and unknown class recognition is constructed by combining AFUCR and SNRSN. Experimental results show that the recognition accuracy of the AFUCR-SNRSN model is the highest in the scenario of a low jamming to noise ratio (JNR). The accuracy is increased by approximately 4–9% compared with other methods on known class interference signal datasets, and the recognition accuracy reaches 99% when the JNR is −6 dB. At the same time, compared with other methods, the false positive rate (FPR) in recognizing unknown class interference signals drops to 9%.

## 1. Introduction

Flying ad-hoc network (FANET) usually undertakes important information collection and transmission tasks in the battlefield environment. Thus, it has high requirements for communication quality. The battlefield environment is expected to become more complex in the future, and the communication between unmanned aerial vehicles (UAVs) occurs in a relatively open environment, which makes it more susceptible to malicious interference. Thus, the accuracy of information transmission and the security rates of communication systems are reduced. Considering the above problems, it is of great research significance to reduce the interference’s influence on the communication quality.

### 1.1. Related Work

To solve the interference influence in open environments, some researchers have proposed advanced interference suppression methods. Lin proposed methods based on beamforming [1,2,3] and RIS [4] to improve the communication quality using interference suppression. These methods have great effects when the interference intensity is relatively low. However, if there is a higher requirement for communication quality, there must be interference recognition methods to achieve interference suppression.

Traditional interference recognition methods mainly include decision tree and support vector machine (SVM) methods. The method based on decision trees extracts distinguishing features. Then, it sets the threshold of the features and classifies the interference signals layer by layer. Luo [5] extracted 19-dimensional features from the time domain, frequency domain and so on. Then, they used PCA to compress the features into nine dimensions and used a decision tree to recognize them. Xu [6] first chose distinguishing skewness features as the root nodes of the decision tree, which classified the signals into four types. To execute further classification, they set a threshold for the features, including the envelope fluctuation parameter, kurtosis, instantaneous amplitude–frequency maximum and frequency stability. SVM can distinguish different interferences by solving the maximum margin hyperplane of the train dataset. This also ensures that the SVM will not misclassify samples of the same class. Yan [7] extracted multi-dimension features including the envelope fluctuation parameter, phase threshold probability and box dimension. They adopted SVM to achieve classification into smart noise interference and real echos. Li [8] first performed a double orthogonal Fourier transform on the signals, extracted the peak slope and set its threshold. After dividing the frequency spectrum diffusion interference, slice reconstruction interference, noise multiplication interference, noise convolution interference, intermittent sampling forwarding interference and real echo into three groups, five-dimensional features such as spectral entropy and cross-correlation coefficients were extracted and input into a trained support vector machine to complete the recognition and classification. Fan [9] extracted 10-dimensional features from the time domain, frequency domain and transform domain. Then, they selected features with better anti-noise performance through their correlations to complete the recognition of various active interferences, such as RF noise, noise amplitude modulation and distance deception. The traditional interference recognition method requires manual feature extraction and the selection of classifiers to classify data according to features, which takes a long time and has limited promise. The recognition accuracy of communication interference signals is still not quite satisfactory under a low jamming to noise ratio (JNR), and the recognition of new types of interference signals in complex battlefield environments is not considered, which means that the network lacks scalability.

In recent years, deep neural networks (DNNs) have been widely used in the fields of image and speech processing and have achieved good results, so many scholars have also applied them to the field of communication interference recognition and research. Zhao [10] improved the LeCun Network (LeNet) proposed by LeCun to complete feature extraction and final classification through two-layer convolution. Su [11] proposed an interference recognition method based on feature parameters, which used one-dimensional convolution to extract features with low sensitivity to noise from interference signals and identify features according to theoretical thresholds. Focusing on a small number of communication interference signal samples, Tang [12] proposed an auxiliary classifier variational auto-encoding generative adversarial network (AC-VAEGAN) model, which combined auxiliary classifiers with automatic variational coding to generate adversarial networks to augment small sample sets, so that despite a lack of samples, the model could still achieve high recognition accuracy. Shao [13] used the one dimension-convolutional neural network (1D-CNN) model to extract features from real signals and imaginary signals and fuse them for comparison.

Yang [14] used the powerful feature extraction ability of the residual neural network (ResNet) to analyze the interference signal and replaced the convolution in ResNet with hole convolution, which effectively improved the feature extraction capability without significantly increasing the number of parameters, and they built a lightweight residual network (LRN). Zhang [15] proposed a multimodal feature fusion method, which first obtained the constellation map by signal processing of the collected interference signal and then used the convolutional neural network (Alex Network, AlexNet) proposed by Alex Krizheveky to extract the features of the constellation map. The complex network was used to extract the features of the original signal, and then the features were fused and identified. Ali Pourranjbar [16] used the channel information occupied by the interference signal for the first time, took the interference channel as input and used the recurrent neural network to analyze the channel interference situation and interference signal to realize the recognition. Liu [17] proposed to apply federated learning to the interference recognition model when the sample size of communication interference signals was small and scattered. The training parameters of different submodels were aggregated and globally optimized through federated learning, and, finally, the effective recognition of interference signals could be realized in a distributed and few-sample situation. Qiu [18] used dilated convolution to expand the characteristics of the receptive field of the model and constructed hybrid dilated convolution (HDC) without a significant increase in the number of parameters. In the neural network model, the time–frequency map of the interference signal was first obtained by short-time Fourier transform (STFT), and then the feature extraction of larger receptive fields was realized through the HDC network to complete the signal recognition. Dong [19] proposed a time–frequency long short-term memory (TF-LSTM) model based on time–frequency analysis. Firstly, the time–frequency analysis was used to pre-process the interference signal, and then the long short-term memory network could fully obtain the characteristics of the correlation bidirectionally, extract the characteristics of the signal and finally complete the recognition. Shen [20] proposed a residual network based on soft fusion (Res-soft). Res-soft utilized multi-node cooperation to achieve data fusion, which helped to recognized interference.

However, the above methods do not pay specific attention to the suppression and processing of noise in the signal, and the network models are simple with few layers, so the accuracy is still unsatisfactory under a low JNR. Moreover, the above methods do not consider that in complex battlefield environments, UAVs are likely to be attacked by unknown interference with the modification of attack methods.

### 1.2. Contributions

**Figure 1:**
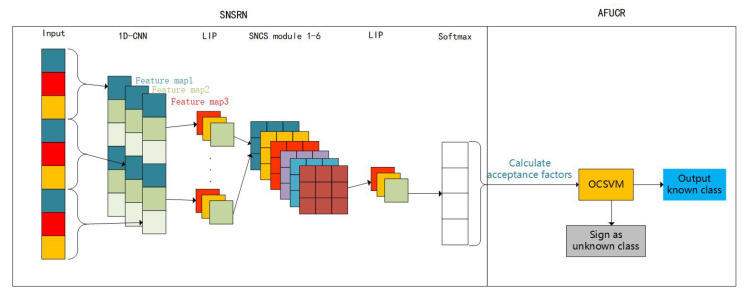
Network model structure.

Motivated by the above problems, a network model based on the improved deep residual shrinkage network is proposed, which is called AFUCR-SNRSN, for known and unknown recognition. The structure of the model is shown in Figure 1. A simple non-local correction shrinkage (SNCS) module is constructed that embeds the correction threshold function operation into the neural network. Thus, the neural network is able to evaluate the rationality of the noise threshold automatically. Meanwhile, a local importance-based pooling (LIP) strategy is introduced to automatically enhance the useful signal features in the downsampling process to further weaken the noise. Moreover, the joint loss function is constructed to further improve the accuracy. According to the above, one simplified non-local residual shrinkage network (SNRSN) model is proposed to complete the task of signal recognition. In addition, after observing and analyzing the confidence distribution of known and unknown interference in the softmax layer, the acceptance factor is proposed. The acceptance factor-based unknown class recognition (AFUCR) and SNRSN are combined to form an AFUCR-SNRSN model with the capacity for both known class classification and new class rejection.

The main contributions of this research are as follows:

(1) We modify the soft thresholding function in the deep residual shrinkage network to avoid eliminating useful features, and a simple non-local module is introduced into the network to evaluate the threshold from both the spatial dimension and channel dimension.
(2) We introduce the LIP strategy into the downsampling process to suppress noise. We propose a joint loss function to allow different features to be “separable between classes and compact within classes”, which can improve the recognition accuracy in low JNR situations.
(3) Focusing on unknown interference recognition in an open set, we propose an acceptance factor based on the confidence distribution difference between known and unknown interference. Using the acceptance factor, unknown interference recognition could be converted into a one-class classification problem and solved.
(4) According to various simulation experiments, it is verified that our method not only can recognize interference under a high JNR, but also retains relatively high accuracy in low JNR situations.

## 2. Model and Formulation

In the study, the proposed model is composed of two parts, SNRSN and AFUCR, which are shown in Figure 1.

(1) SNRSN: Based on the deep residual shrinkage network (DRSN) [21], the soft threshold function is corrected to avoid eliminating useful features of the signal. Then, a simple non-local module is introduced to evaluate the threshold from both the spatial and channel dimensions, which improves the DRSN shrinkage module. This method uses the LIP strategy to enhance the recognition features and weaken the noise features. Moreover, the center loss function and the cross-entropy loss function are combined to construct a joint loss function, which is used to train the model.
(2) AFUCR: Through analyzing the confidence distribution difference output from the softmax layer between known and unknown class interference, this method proposes an acceptance factor. Unknown interference recognition can be converted into a one-class classification problem and solved by calculating samples’ acceptance factors.

### 2.1. SNRSN

**Figure 2:**
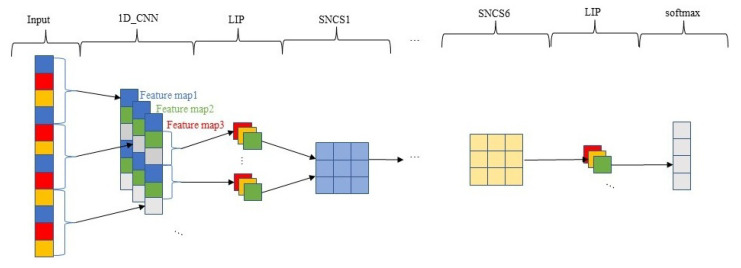
SNRSN model structure.

Based on DRSN, the SNCS module is used to replace the residual shrinkage module in DRSN. SNRSN also introduces the LIP strategy and constructs a joint loss function. The structure of SNRSN is shown in Figure 2. The concrete parameters of each network layer are shown in Table 1. The first and second parameters in the Layer column’s parentheses denote the number and the size of the Conv kernel, while the third parameter refers to the step size of the Conv kernel movement. If this parameter is missing, it means that the step size is the default value of 1; “/2” means that the step size is 2.

#### 2.1.1. SNCS Module

A soft threshold is widely used in the field of traditional signal denoising, such as wavelet denoising. Wavelet denosing mainly consists of three steps: wavelet decomposition, soft thresholding and wavelet reconstruction. Wavelet decomposition involves transforming the original signal into a decomposable scale. Under this scale, coefficients close to 0 are considered unimportant and may be seen as noise. Soft thresholding is used to compress this signal to 0. Its formula is shown in (1), where *x* represents the input feature and τ represents the threshold.

(1) $$\begin{array}{c} y=\left\{\begin{array}{cc} x-\tau & x>\tau \\ 0 & -\tau \leq x\leq \tau \\ x+\tau & x<-\tau \end{array}\right. \end{array}$$

The usage of soft thresholding is to set coefficients that are less than the threshold to 0 (filter noise) and shrink the others. A soft threshold can avoid the discontinuity problem of a hard threshold function. From (1), the derivative of the soft threshold function can be calculated as in (2).

(2) $$\begin{array}{c} \frac{\partial y}{\partial x}=\left\{\begin{array}{cc} 1 & x>\tau \\ 0 & -\tau \leq x\leq \tau \\ 1 & x<-\tau \end{array}\right. \end{array}$$

However, the traditional method of threshold selection requires multiple attempts and a lot of expertise in signal processing. In 2020, Zhao proposed the DRSN [21] model for strong noise signal data processing. DRSN added soft thresholding as a shrinkage module to the residual network. Thus, the threshold could be adjusted adaptively, which is suitable for handling interference signal recognition problems under a low JNR.

**Figure 3:**
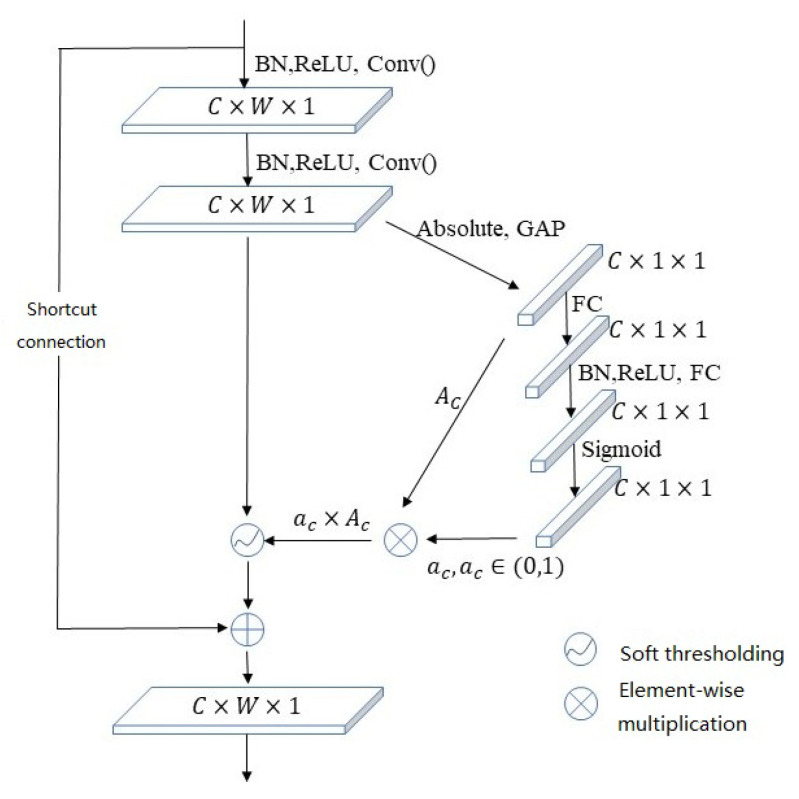
DRSN shrinkage module.

The DRSN shrinkage module is shown in Figure 3. The structure of this module is similar to the channel attention module. The first step is obtaining the absolute value, which is denoted by $A_{c}$, of each channel’s feature map. Then, it performs global average pooling on the feature map’s channel dimension. The generated 1-D vector next passes through two fully connected layers and performs a sigmoid process. The output is denoted as $a_{c}$. Channel threshold $\tau_{a}$ is calculated by $a_{c}*A_{c}$. Then, the module performs soft thresholding on each of the feature map’s channels. The denoising is achieved by zeroing unimportant channel features.

(1) Threshold Correction

**Figure 4:**
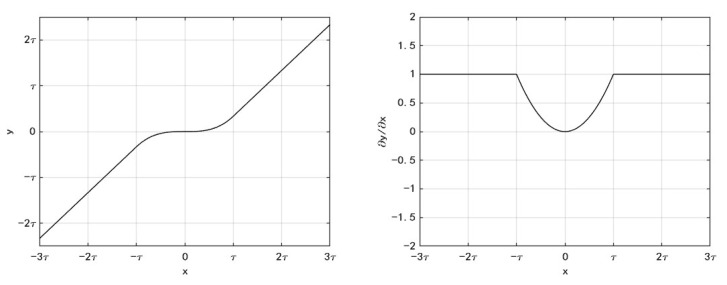
Correction threshold function (**left**) and its derivative (**right**).

As shown in (3), soft thresholding in each channel directly zeros all features below the threshold, which may eliminate a few useful features other than noise, thereby reducing the signal recognition accuracy [22]. To solve this problem, the soft threshold function is modified. Assuming that the threshold is τ, the modified threshold function is still an odd function and can be defined as y=f(x). When −τ≤x≤τ, $f\left(x\right)=ax^{3}+bx$. When x>τ, f(x)=x+c. When x<τ, f(x)=x−c. To ensure that the correction threshold function is continuous at the threshold, $a\tau^{3}+b\tau =\tau +c$. At the same time, to ensure that the correction threshold function is derivable at the threshold, $f^{\prime }\left(\tau \right)=1$, $f^{\prime }\left(-\tau \right)=1$. According to the above calculations, $a=\frac{1}{3\tau^{2}}$, b=0, $c=-\frac{2}{3}\tau$. The final correction threshold function is shown in (3), and the function image is shown in Figure 4. The derivative solved for (3) is shown in (4). Its function image is also shown in Figure 4.

(3) $$\begin{array}{c} y=\left\{\begin{array}{cc} x-\frac{2}{3}\tau & x>\tau \\ \frac{1}{3\tau^{2}}x^{3} & -\tau \leq x\leq \tau \\ x+\frac{2}{3}\tau & x<-\tau \end{array}\right. \end{array}$$

(4) $$\begin{array}{c} \frac{\partial y}{\partial x}=\left\{\begin{array}{cc} 1 & x>\tau \\ \frac{1}{\tau^{2}}x^{2} & -\tau \leq x\leq \tau \\ 1 & x<-\tau \end{array}\right. \end{array}$$

Equations (3) and (4) show that the modified threshold function does not completely zero the feature when the absolute value is smaller than the threshold, and some useful signal information is still retained. While the noise information is greatly suppressed, it is not completely eliminated. The derivative is always between 0 and 1, which can prevent gradient disappearance and explosion problems during the training process.

(2) Simple Non-Local Module

DRSN is similar to the channel attention module in threshold evaluation, focusing only on channel dimension information and ignoring information in the spatial dimension. Therefore, a simple non-local module [23] is introduced to replace the global average pooling in the original module. This module comprehensively considers the spatial dimension information and channel dimension information, so that the threshold selection is more accurate.

**Figure 5:**
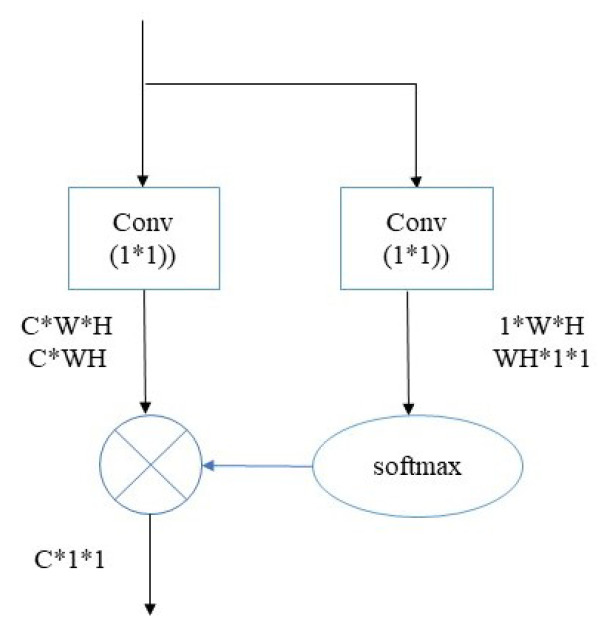
Simple non-local module.

The simple non-local module refers to the idea of non-local means denoising. It is a module based on the spatial attention mechanism. The features that have a large distance from each other can be related by calculating the correlation of their positions on the feature map. The structure of the simple non-local module is shown in Figure 5.

This module first compresses the input feature map with dimension C×W×H in the channel dimension so as to obtain a single feature map with dimension 1×W×H, which contains the information of all positions in the input feature map to make full use of the global information. The correspondence between each position feature and all other position features in the feature map is calculated by the softmax function to form an attention map. Then, the attention map is multiplied at the element level with the input feature map with dimension C×W×H, and, finally, all elements in the feature map in each channel are added to obtain the global context relationship with dimension C×1×1. The last two steps are changed to the matrix multiplication of C×WH and WH×1×1 in order to simplify the calculation. The calculation is shown in (5), where *x* represents the input and φ() represents the linear change matrix.

(5) $$\begin{array}{c} \begin{array}{cc} s\left(x\right)=\sum_{i=1,j=1}^{W,H}\frac{exp(\varphi \left(x_{ij}\right))}{\sum_{a=1,b=1}^{W,H}exp\left(\varphi \left(x_{ab}\right)\right)}x_{ij} & x\in R^{W,H,C} \end{array} \end{array}$$

**Figure 6:**
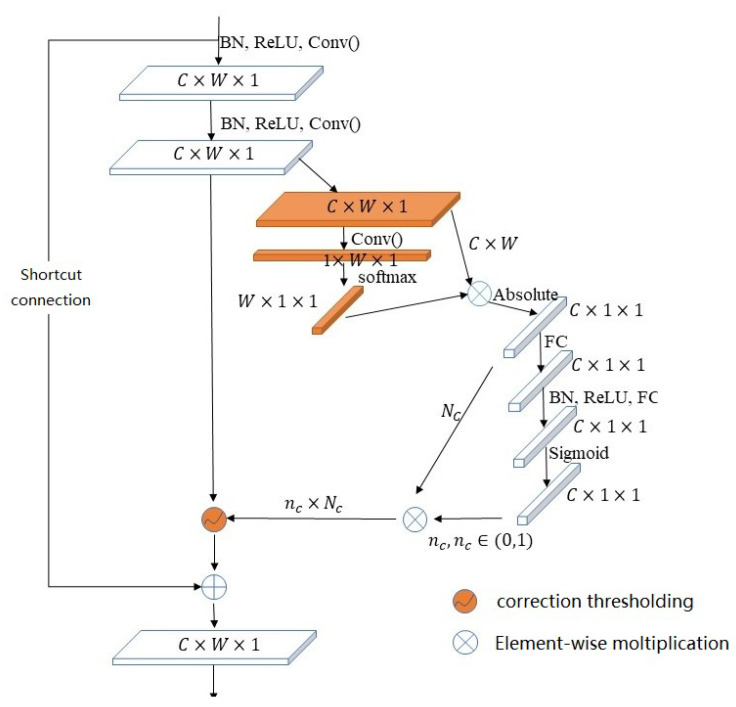
SNCS module.

Replacing the global average pooling in the DRSN shrinkage module with the simple non-local module can fully utilize the global context information. Combined with spatial information to obtain the long-term dependency between features, it can evaluate the threshold more concretely. Then, replacing the soft threshold function with the proposed correction threshold function, the simple non-local correction shrinkage (SNCS) module is proposed. The structure of the SNCS module is shown in Figure 6. The left branch retains the absolute value of the weight obtained from the global spatial information, which is recorded as $N_{c}$. The right branch interacts with messages across channels to extract the relationship between each channel and obtain the scale parameters of different channels. Then, it obtains the scale parameters $n_{c}$ from the fusion of channel and spatial information. Finally, the output of the left and right branches is multiplied to obtain the threshold $\tau_{n}$ of each channel feature mapping, calculated as in (6).

(6) $$\begin{array}{c} \tau_{n}=N_{c}n_{c} \end{array}$$

Assume that the input dimension is $I^{C\times W\times H}$. In the module, first, two convolution, ReLU and batch normalization operations are executed to obtain a feature map with dimension $M^{C\times W\times H}$. Then, the threshold $\tau^{C\times 1\times 1}$ of a set of different channels is obtained by the shrinkage module. The threshold and $M^{C\times W\times H}$ are thresholded according to the modified threshold function, so the denoising feature map $T^{C\times W\times H}$ is obtained. Finally, the input and the output after shrinkage are added by identity connection to obtain the output $O^{C\times W\times H}$, calculated as (7).

(7) $$\begin{array}{c} O^{C\times W\times H}=I^{C\times W\times H}+T^{C\times W\times H} \end{array}$$

#### 2.1.2. LIP Strategy

After feature extraction by convolution operations, the quantity of parameters is usually large. Thus, it is necessary to pass through the pooling layer for downsampling, which is a lossy process. The method adopted in classical neural networks is to use average pooling or max pooling. Average pooling assumes that features in all positions are equally important. It does not distinguish features belonging to useful signals or noise. Max pooling assumes that the max value is the most representative one. Thus, it retains the max value but abandons others. These two assumptions are not always correct. It may cause useless noise features to be retained and useful signal features to be abandoned.

A new pooling strategy, LIP [24], is introduced to solve this problem. LIP believes that, in the downsampling process, the contribution of neighboring features is generally different, and the discriminating nature of some location features is stronger than that of other neighboring features. Thus, it is necessary to assign different importance weights to automatically enhance the recognition features and weaken the noise features during the downsampling process.

Given the input feature map *I* and the kernel index set *C* with a relative sampling position of (Δx,Δy) in a pooling window, the starting position in the sliding window is (x,y) and the corresponding output position is $\left(x^{\prime },y^{\prime }\right)$; then, the pooling calculation process is as shown in (8). Let F(I) represent the attention map, which has the same dimension as *I*.

(8) $$\begin{array}{c} O_{x^{\prime },y^{\prime }}=\frac{\sum_{(\Delta x,\Delta y)\in C}F{\left(I\right)}_{x+\Delta x,y+\Delta y}I_{x+\Delta x,y+\Delta y}}{\sum_{(\Delta x,\Delta y)\in C}F{\left(I\right)}_{x+\Delta x,y+\Delta y}} \end{array}$$

LIP generates an importance graph by using a learnable fully convolutional network *g*. To make the importance weights non-negative and easy to optimize, the exp() operation is added on *g*, which means that F(I)=exp(g(I)). The pooling calculation process of LIP can be expressed as in (9).

(9) $$\begin{array}{c} O_{x^{\prime },y^{\prime }}=\frac{\sum_{(\Delta x,\Delta y)\in C}exp{\left(g\left(I\right)\right)}_{x+\Delta x,y+\Delta y}I_{x+\Delta x,y+\Delta y}}{\sum_{(\Delta x,\Delta y)\in C}exp{\left(g\left(I\right)\right)}_{x+\Delta x,y+\Delta y}} \end{array}$$

#### 2.1.3. Joint Loss Function

Due to the strong randomness of the frequency of interference signals and the different noise content in the same category in complex environments, the characteristics of similar signals have a large gap within the class. Therefore, it is worth considering that in the feature space, the interference signals are not only separable between classes but also compact within the class. This modification can obtain a more robust judgment result when the range of similar signals is large. In this case, the center loss function [25] and the cross-entropy loss function are introduced for the joint training of the network.

Center loss can constrain the distances between sample features in the feature space so that the distance between each sample feature and the average features of the same class is small enough. It can achieve compactness within the class. The calculation process is as shown in (10), where m represents the number of samples, $x_{i}$ demonstrates the extracted feature of the *i*-th sample and $C_{y_{i}}$ represents the center point of all sample features in the corresponding category of sample *i*.

(10) $$\begin{array}{c} L_{C}=\frac{1}{2}\sum_{i=1}^{m}{∥x_{i}-C_{y_{i}}∥}_{2}^{2} \end{array}$$

The cross-entropy loss function is calculated as in (11), where n represents the number of categories, $t_{i}$ represents the label value of the *i*-th category and $p_{i}$ represents the predicted probability of the *i*-th category.

(11) $$\begin{array}{c} L_{CE}=-\sum_{i=1}^{n}t_{i}ln\left(p_{i}\right) \end{array}$$

The joint loss calculation is shown in (12), where λ represents the weight of the center loss function.

(12) $$\begin{array}{c} L=L_{CE}+\lambda L_{C} \end{array}$$

### 2.2. AFUCR

**Figure 7:**
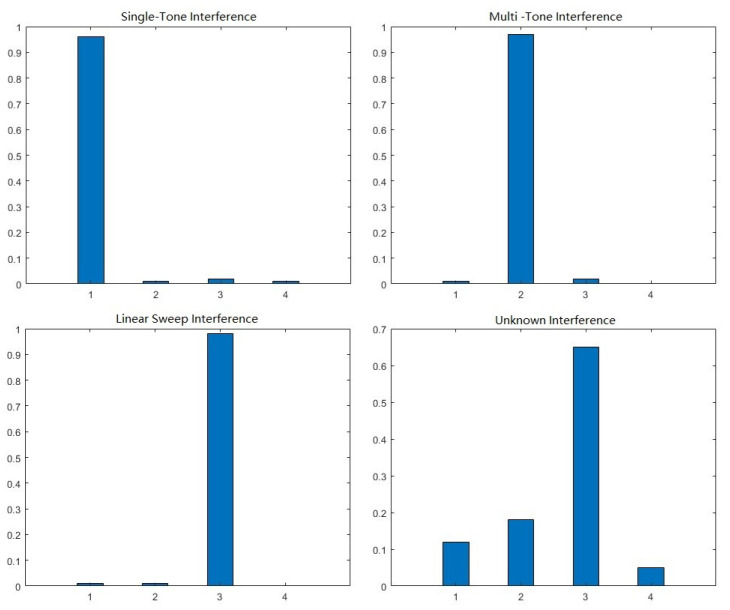
Confidential distribution prediction of different interference signal.

In the −8 dB JNR situation, Figure 7 shows the softmax output from SNRSN, which takes three known interference signals and one unknown interference signal as input. Moving through the SNRSN, even the unknown interference signal can obtain a relatively high-confidence prediction and be classified as one of the known signals. This error information will lead UAVs to make wrong anti-interference decisions, and unknown data cannot be recorded for post-processing.

By observing the confidence distribution prediction in Figure 7, we can see that the confidence prediction of known interference is extremely close to the real class. Meanwhile, the unknown class interference signal is relatively uniform and smooth. Therefore, it is considered that the output confidence distribution can be used to distinguish between known class signals and unknown class signals, and the smoothness of the confidence prediction distribution is defined as the acceptance factor, calculated as in (13).

(13) $$\begin{array}{c} acceptancefactor=\frac{maxS(x)}{1-maxS(x)} \end{array}$$

**Figure 8:**
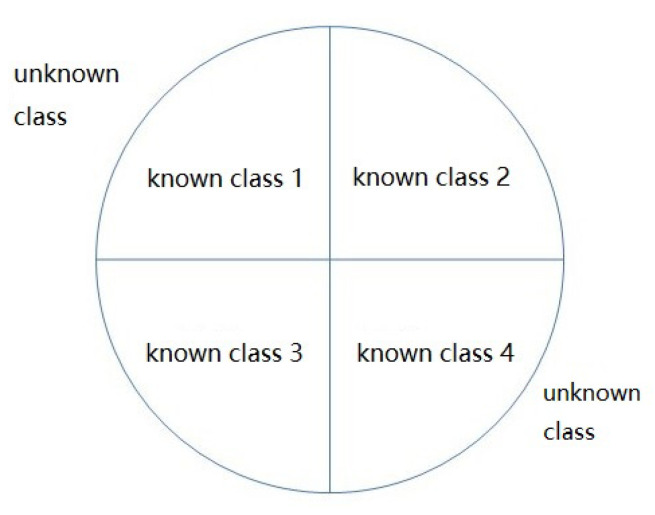
Difference between known and unknown interference.

In the training process, only training samples of known class signals can be accessed, so the problem is converted into a one-class classification problem, which means that a hypersphere is trained in the training phase to contain all training samples. Meanwhile, in the testing phase, the samples inside the hypersphere are recognized as known classes, and the outside samples are marked as unknown classes. The simulation of the samples’ distribution in the hypersphere is shown in Figure 8.

According to the above characteristic, one-class vector machine (OCSVM), a classical class of support vector machines, is selected as the classification training model. Using a kernel function, the origin point can be defined as the only error sample. Then, the unknown interference recognition problem can be converted into a classical SVM problem. The classification hyperplane’s representation is shown in (14), where *x* is the train samples that come from the acceptance factors output from the softmax layer in the SNRSN train process, and φ(x) is the nonlinear mapping function.

(14) $$\begin{array}{c} f\left(x\right)=\omega^{T}\varphi \left(x\right)-\rho =0 \end{array}$$

To maximize the distance between outliers and the classification hyperplane, the solution of SVM can be transformed into a quadratic programming problem as in (15), where δ is the slack variable, *l* is the number of the samples and *v* is the penalty parameter. Moreover, v∈[0,1].

(15) $$\begin{array}{cc} & min\frac{1}{2}{∥\omega ∥}^{2}+\frac{1}{vl}\sum_{i=1}^{l}\delta_{i}-\rho \\ & s.t.\;\left(\omega *\varphi \left(x_{i}\right)\right)\geq \rho -\delta_{i}\\ & \delta_{i}\geq 0\;i=1,2,\ldots ,l \end{array}$$

Based on (15), the Lagrange function can be constructed as in (16).

(16) $$\begin{array}{c} L\left(\omega ,\delta ,\rho ,\alpha ,\gamma \right)=\frac{1}{2}{∥\omega ∥}^{2}+\frac{1}{vl}\sum_{i=1}^{l}\delta_{i}-\rho -\sum_{i=1}^{l}\gamma_{i}\delta_{i}-\sum_{i=1}^{l}\alpha_{i}\left[\omega^{T}\varphi \left(x_{i}\right)-\rho +\delta_{i}\right] \end{array}$$

According to the Lagrange function’s duality, the dual problem of (16) is (17).

(17) $$\begin{array}{c} \underset{\alpha }{max}\;\underset{\omega ,\;\rho }{min}\;L\left(\omega ,\delta ,\rho ,\alpha ,\gamma \right) \end{array}$$

Calculating the partial derivative of (16) and letting the partial derivative be equal to zero results in (18).

(18) $$\begin{array}{cc} & \omega =\sum_{i=1}^{l}\alpha_{i}\varphi \left(x_{i}\right)\\ & \sum_{i=1}^{l}\alpha_{i}=1\\ & \alpha_{i}=\frac{1}{vl}-\gamma_{i} \end{array}$$

Substituting (18) into (16) leads to (19).

(19) $$\begin{array}{c} L\left(\omega ,\delta ,\rho ,\alpha ,\gamma \right)=-\frac{1}{2}\sum_{i=1}^{l}\sum_{i=1}^{l}\alpha_{i}\alpha_{j}k\left(x_{i},x_{j}\right) \end{array}$$

In this situation, the dual problem of (15) can be represented as in (20), where $k(x_{i},x_{j})$ represents the kernel function used to project raw data to a high-dimensional space. This kernel function can be represented as $\langle \varphi \left(x_{i}\right),\varphi \left(x_{j}\right)\rangle$.

(20) $$\begin{array}{cc} & min\frac{1}{2}\sum_{i=1}^{l}\sum_{i=1}^{l}\alpha_{i}\alpha_{j}k{\left(x_{i},x_{j}\right)}^{l}\delta_{i}-\rho \\ & s.t.\;\sum_{i=1}^{l}\alpha_{i}=1\\ & 0\leq \alpha_{i}\leq \frac{1}{vl}\;i=1,2,\ldots ,l \end{array}$$

After solving (20) and obtaining the optimal solution α, ρ and ω can be represented as in (21).

(21) $$\begin{array}{cc} & \omega =\sum_{i=1}^{l}\alpha_{i}\varphi \left(x_{i}\right)\\ & \rho =\langle \omega \varphi (x_{i})\rangle \\ & s.t.\;\alpha_{i}\in \left(0,\frac{1}{vl}\right) \end{array}$$

Substitute the solved result in (21) into (14), and the classification hypersphere can be constructed and the OCSVM problem is solved. AFUCR consists of the calculation of the acceptance factor and OCSVM. Combining AFUCR and SNRSN, the complete AFUCR-SNRSN model is constructed.

## 3. Algorithm Process

**Figure 9:**
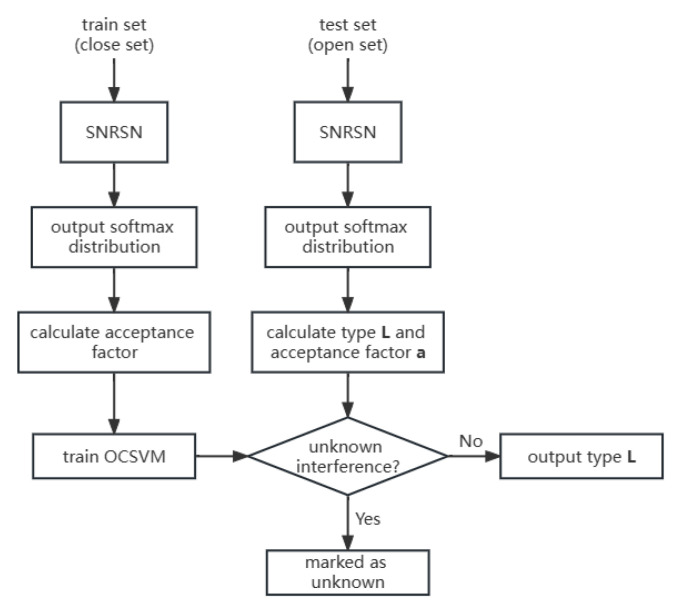
Process of interference signal recognition.

The training and test process of the algorithm is shown in Figure 9. The process can be described in several steps as follows.

1. We use the 1-D CNN to transform the input signal’s type into 2-D and extract the basic features of the signal. Then, the LIP layer is used for downsampling according to (9) to decrease the quantity of the parameters and retain the useful features more precisely.
2. After LIP, features are processed by 6 SNCS modules. The operation of a single SNCS module can be mainly described in the following two steps: (1) using a spatial attention mechanism with multi-channel information fusion and SNM to calculate the correlation between each feature in different positions according to (5); (2) calculating the threshold according to (6) using the correlation from the last step. Then, we adopt the threshold in the correction soft threshold function (3) to filter noise and useless features.
3. Outputs of SNCS modules are processed by LIP and softmax to generate a temporary recognition result. According to (13), the sample’s acceptance factors can be calculated using the output from softmax. The OCSVM is then trained using the set of acceptance factors.
4. The test samples are fed into the trained SNRSN to obtain the confidence distribution and classification results of the softmax layer. According to the output results of the softmax layer, the acceptance factor is calculated and input into the trained OCSVM model. If the output category is “+1”, the previously obtained classification result is directly output; otherwise, the sample is marked as an unknown class and added to the unknown sample library.

## 4. Dataset and Experimental Setup

The experimental dataset is generated by Matlab2022, the sampling frequency of the interference signal $f_{s}$ is set to 10 MHz, and the number of sampling points is 1024. The dataset contains six types of signals: single-tone interference, multi-tone interference, linear sweep interference, time-domain Gaussian pulse interference, noise amplitude modulation (AM) interference and noise frequency modulation (FM) interference. Each dataset is randomly generated according to the parameters of the mathematical model, and the parameter ranges are shown in Table 2. Single-tone interference, multi-tone, linear sweep and time-domain Gaussian pulse are marked as 1, 2, 3 and 4, respectively. Noise AM and noise FM interference are uniformly set to −1 as unknown interference signals. The known class training set consists of four known signals in the range of −16 to 0 dB with a 2 dB JNR interval. In total, 8000 training samples are generated for each type of signal at each JNR to train the model. The test set consists of six signals in the range of −16 to 0 dB with a 2 dB JNR interval. Each type of signal generates 2000 test samples under every JNR to test the performance of the model. Unknown interference signals’ JNRs are in the range of −16 to 0 dB with a 2 dB JNR interval. In total, 1000 samples are generated for each type of unknown interference at each JNR to test the model.

The training and testing of the network model involve a large number of network parameters, and the main parameters and initial settings are as follows: the batch size is initially set to 128, the number of iterations is set to 50 and the learning rate mainly affects the learning speed in the network, which is usually initially set to a small value of 0.001 and adaptively adjusted using the Adam optimization algorithm. Differences in the dimensions and values of the variables may cause incomparability between different signals. To solve this problem, the signal amplitude is normalized, which can avoid unnecessary interference caused by the signal strength. The normalization method is shown in (22), where r(n) represents the amplitude of the n-th sampling point, and l represents the number of sampling points.

(22) $$\begin{array}{c} r^{\prime }\left(n\right)=\frac{r(n)}{\sqrt{\frac{1}{l}\sum_{m=1}^{l}{\left|r\left(m\right)\right|}^{2}}} \end{array}$$

Through linear transformation, whole data are transformed into the distribution whose mean is 0. At this time, the value of the data represents the data’s off-center degree. The centralization method is shown in (23).

(23) $$\begin{array}{cc} & r^{\prime }\left(n\right)=r\left(n\right)-\mu \\ & where\;\mu =\frac{1}{l}\sum_{m=1}^{l}r\left(m\right) \end{array}$$

## 5. Performance

To evaluate the accuracy and potential of the proposed AFUCR-SNRSN model, an ablation experiment is applied to SNRSN to check each module’s performance in recognizing interference. Then, the classic SVM, 1D-CNN in [13], LRN in [14], Res-soft in [20] and SNRSN are used to compare their performance in terms of interference signal recognition on a close set. Lastly, each method above is compared with the AFUCR-SNRSN model on an open set. Through the analysis of the results, the possibilities and effectiveness of the proposed model can be proven.

### 5.1. Determination of Weight of Center Loss Function

**Figure 10:**
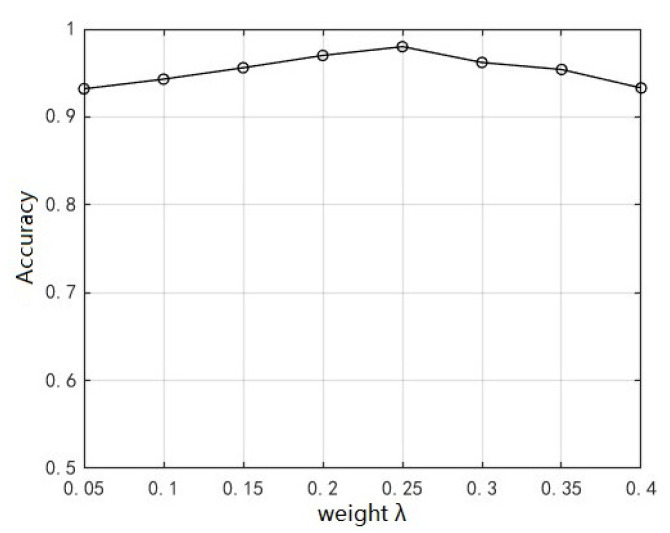
Recognition accuracy variation with weight of center loss function.

As mentioned in Section 2.1.3, the center loss function and cross-entropy loss function are introduced to construct a joint loss function. The weight of the center loss function in (12) needs to be determined in an experiment. Through multiple tests, the network recognition accuracy is the highest when the weight is 0.25, as shown in Figure 10. Thus, it is set to 0.25 in the following experiments.

### 5.2. Ablation Experiment

**Figure 11:**
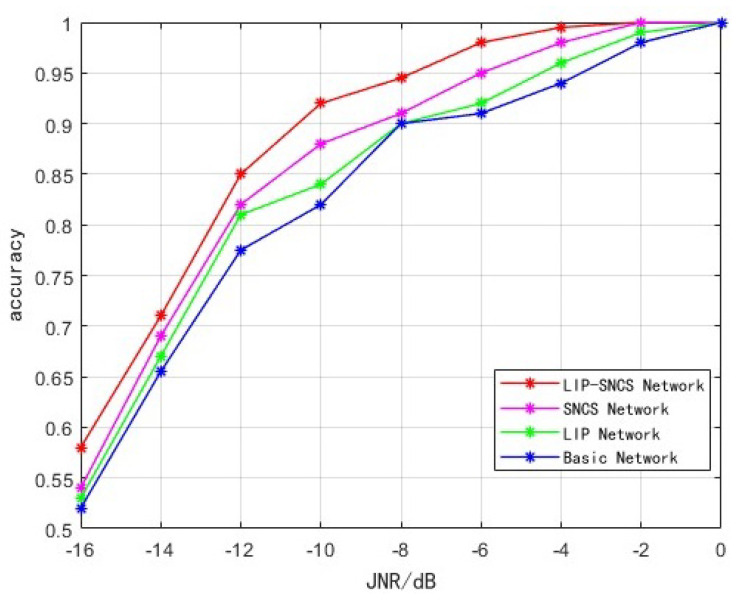
Accuracy of different modules.

(a) In order to test the impact of the introduction of the SNCS module and LIP pooling strategy on the network recognition rate, the cross-entropy loss function is uniformly adopted. The network with maximum pooling and the original residual shrinkage module is recorded as the basic network. The pooling policy in the basic network replaced with the LIP is recorded as the LIP network. The residual shrinkage module in the basic network replaced by the SNCS module is recorded as the SNCS network. The proposed network is denoted as the LIP-SNCS network. The recognition accuracy at different JNRs is shown in Figure 11.

As can be seen from Figure 11, the recognition accuracy of other networks is improved compared with the basic network.

Specifically, compared with the base network, the accuracy of the LIP network is improved by approximately 2% in the range of −16–0 dB. This indicates that the maximum pooling replaced by the LIP can suppress noise and strengthen the discriminating features during the downsampling process, so that useful information can be extracted under a low JNR.

The SNCS network improves the accuracy by approximately 5% compared to the basic network in the range of −16–0 dB. This indicates that the SNCS module can pay more attention to the global information compared with the original residual shrinkage module, obtain long-range dependencies and select the threshold more accurately. The modified threshold function also avoids the filtering of useful features, further improving the recognition accuracy. The SNCS network has higher recognition accuracy than the LIP network, because LIP pooling only occurs twice in the whole model. Moreover, the first downsampling occurs at a shallow network level where feature extraction is not sufficient, while the SNCS module in the network suppresses noise and pays attention to global information in the deeper network layer. Thus, the recognition accuracy is higher.

The LIP-SNCS network replaces the original residual shrinkage module with the SNCS module and the pooling strategy with LIP, which has the best overall performance. The accuracy is improved by approximately 7%, and, when at −4 dB, the recognition accuracy reaches more than 99%.

**Figure 12:**
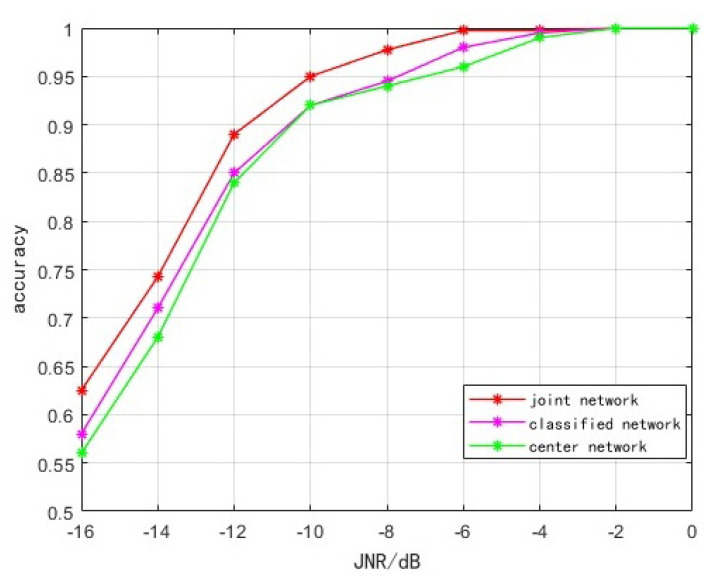
Accuracy of different losses.

(b) In order to analyze the impact of the loss function on the network performance, the network using the cross-entropy loss function is recorded as the classified network, the network using the center loss function is recorded as the center network and the network using the joint loss function is recorded as the joint network. The recognition accuracy under different JNRs is shown in Figure 12.

It can be seen from Figure 12 that in the range of −16–0 dB, the accuracy of the classification network and the center network is very close. The accuracy of the classification network is approximately 1–2% higher compared with the center network, because the cross-entropy loss function pays more attention to the class information of the sample, while the center loss function is more concerned with compactness within the class. Therefore, it is inferior to the classification network in recognition accuracy.

Compared with the classification network and center network, the recognition of joint network accuracy improvement is approximately 4%, indicating that the joint guidance can improve the performance of the network.

### 5.3. Closed Set Test

**Figure 13:**
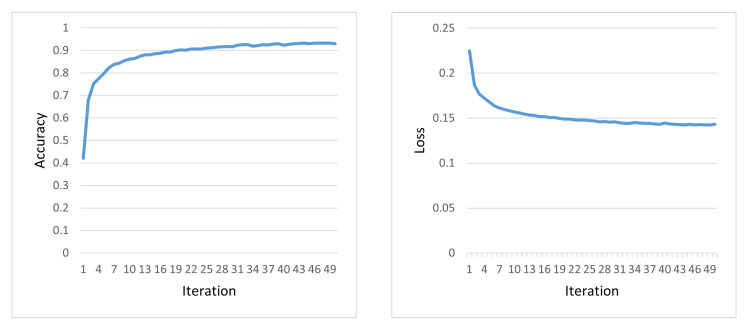
Accuracy and loss variations with iterations.

First, the model is trained by a known class training set. The recognition accuracy and loss in training process are shown in Figure 13. In the first 30 iterations, the accuracy of the model increases and the loss of the model decreases continuously. This shows that the model’s ability to classify increases gradually. After 30 iterations, both the accuracy and loss of the model become stable and convergent without obvious fluctuations. This proves that the network has stability and does not display overfitting.

**Figure 14:**
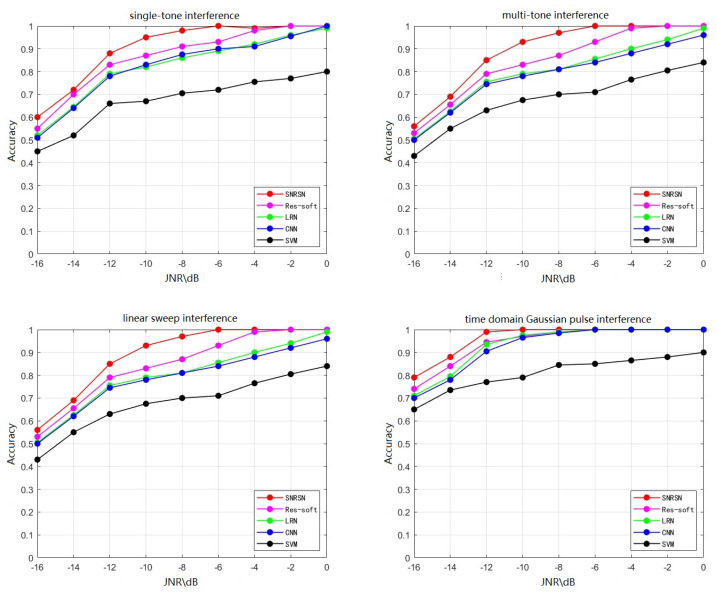
Comparison of different networks.

The proposed SNRSN network model is compared with the traditional SVM, 1D-CNN, LRN and Res-soft on the known interference test set. The results are shown in Figure 14.

It can be seen from Figure 14 that the recognition accuracy of the four methods improves with the increase in the JNR during the test, indicating that the decrease in noise content makes it easier to extract the features of the interference signal.

In particular, the SNRSN network generally outperforms the other three methods in recognizing interference signals under the condition of each JNR. In the strong noise environment of −16 dB, the SNRSN network model has recognition accuracy of more than 55% for the four interference signals. As for the time-domain Gaussian pulse interference, the recognition accuracy is more than 80%, because it behaves very differently from the other three signals in the time-domain waveform. Multi-tone interference is superimposed by multiple single-tone interference so the waveform is more complex and the recognition accuracy is slightly lower than that of other interference signals. When the JNR reaches −6 dB, the SNRSN recognition accuracy for all four types of interference reaches 99%.

The SVM method relies on manual feature extraction. Thus, in low JNR situations, its accuracy is around 40%. Because 1D-CNN and LRN only consist of two Conv Layers, which leads to low feature extraction ability, they cannot extract the features of interference signals in low JNR situations. Their accuracies are close and relatively low. Res-soft fully utilizes the data of multiple nodes to evaluate and uses soft fusion to judge. Thus, its recognition accuracy is higher compared to CNN and LRN. However, it does not focus on noise processing and suppression. The extracted features include many redundant and wrong features, which leads to relatively low accuracy in low JNR situations.

The proposed SNRSN executes a denoising process during feature extraction. Compared with Res-soft, its recognition accuracy increases by approximately 2–10% in the JNR range of −16 to −6 dB. This proves that SNRSN focuses more on useful features in the signal and suppresses noise to make the network perform more stably. SNRSN has robustness and noise immunity. Each model’s recognition accuracy on each type of interference is shown in Table 3. It proves that the proposed model has better performance comprehensively.

**Figure 15:**
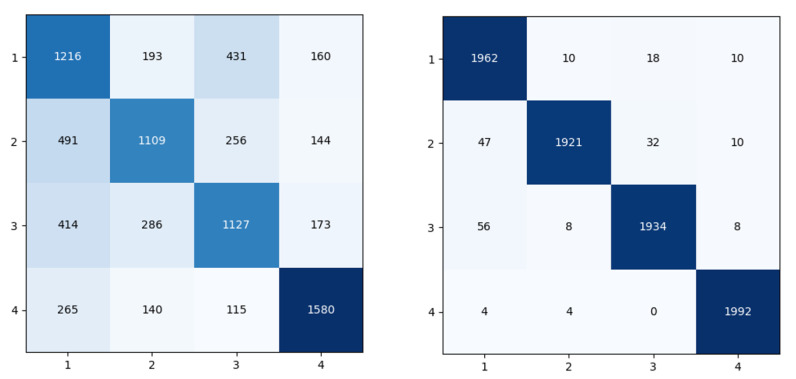
Confusion matrix in −16 dB (**left**) and −8 dB (**right**) JNR.

To analyze the classification results on known interference, the confusion matrix under a −16 dB and −8 dB JNR is adopted, as shown in Figure 15. In the −16 dB JNR, it can be seen from Figure 15 that single-tone interference may be recognized as multi-tone interference or linear sweep interference and sometimes time-domain Gaussian pulse interference. Because the waveform of multi-tone interference overlaps with that of single-tone interference, multi-tone interference is often misrecognized as single-tone interference and sometimes linear sweep interference. Linear sweep interference is misrecognized as single-tone interference most. Many time-domain Gaussian pulse interferences are misrecognized as single-tone interference. Fewer instances are misrecognized as other types. In the −8 dB JNR, most of the signals are not misrecognized.

### 5.4. Open Set Test

In order to test the scalability of each method for interference signal testing, the known interference signal set and the unknown class interference signal set are combined to form an open test set. The CNN, LRN, TF-LSTM, SNRSN and AFUCR-SNRSN models are tested, and the model results are evaluated using two indicators, which are well-accepted metrics in intelligent recognition to compare model performance [26,27]: the true positive rate (TPR) and the false positive rate (FPR), calculated as in (24) and (25). True positive (TP) represents the number of known class samples that are correctly recognized as a known class. False negative (FN) indicates the number of known class samples that are misrecognized as an unknown class. False positive (FP) represents the number of unknown class samples that are misrecognized as a known class. True negative (TN) indicates the number of unknown samples that are correctly recognized as an unknown new class.

(24) $$\begin{array}{c} TPR=\frac{TP}{TP+FN} \end{array}$$

(25) $$\begin{array}{c} FPR=\frac{FP}{FP+TN} \end{array}$$

**Figure 16:**
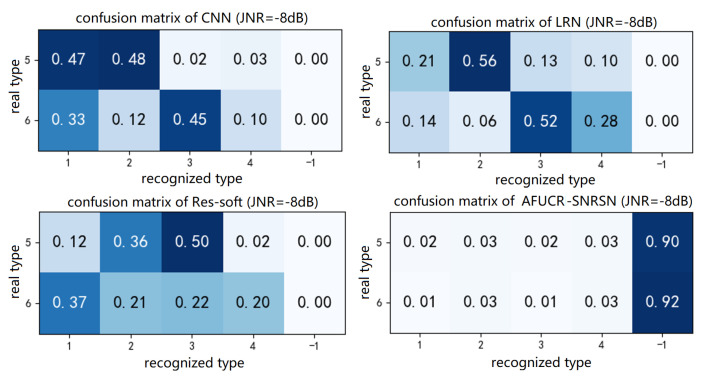
Confusion matrix of unknown interference.

The experimental results are shown in Table 4. The confusion matrix of unknown interference is shown in Figure 16. Combining the figure and the table, it can be seen that the FPR of CNN, LRN, TF-LSTM and SNRSN is always 1, indicating that there is no rejection function at all for unknown types of interference signals. Meanwhile, for the AFUCR-SNRSN model, it is 0.09, which means that it can distinguish between unknown and known interference signals.

## 6. Conclusions

To improve the recognition performance of communication interference signals in a strong noise environment, one SNCS model that embeds the modified threshold function and a simple non-local module into the residual shrinking network is proposed. The LIP pooling strategy and the joint loss function are also introduced to obtain better performance. The SNRSN model can obtain an approximately 4–9% increase in recognition accuracy compared to other methods; moreover, the accuracy in recognizing four known types of interference signals under the JNR of −6 dB reaches 99%.

Based on the analysis of the confidence distribution of known and unknown class signals in softmax, the acceptance factor is proposed to distinguish between known and unknown classes. The AFUCR-SNRSN model combined with AFUCR and SNRSN reduces the FPR to 0.09, which means that it has the capacity for both known class recognition and new class rejection.

Furthermore, future work will focus on using existing models for transfer learning to recognize more interference signals.

## Figures and Tables

**Table 1:** Network’s parameters.

Output	Layer	Activation Function
1024 × 1	Input	-
16 × 1024 × 1	Conv (16,3,/2)	ReLU
16 × 1024 × 1	LIP	-
16 × 1024 × 1	SNCS (16,3)	ReLU
16 × 1024 × 1	SNCS (16,3)	ReLU
32 × 1024 × 1	SNCS (32,3)	ReLU
32 × 1024 × 1	SNCS (32,3,/2)	ReLU
64 × 1024 × 1	SNCS (64,3)	ReLU
64 × 1024 × 1	SNCS (64,3,/2)	ReLU
64 × 1	LIP	-
4	FC	softmax

**Table 2:** Signal parameters.

Label	Signal	Parameter	Value Range
1	Single-tone	Frequency	[1/20, 1/10] $f_{s}$
2	Multi-tone	Frequency	[1/20, 1/10] $f_{s}$
3	Linear sweep	Sweep period	[1/50, 1/30] (unit: s)
		Sweep bandwidth	[1/20, 1/10] $f_{s}$
4	Time-domain Gaussian pulse	Duty cycle	[1/40, 1/20]
−1	Noise AM	Interference frequency	[1/20, 1/10] $f_{s}$
−1	Noise FM	Interference frequency	[1/20, 1/10] $f_{s}$

**Table 3:** Accuracy in interference recognition.

Model	Single-Tone	Multi-Tone	Linear Sweep	Time-Domain Gaussian Pulse	Comprehensive Accuracy
SVM	0.67	0.65	0.68	0.81	0.7025
CNN	0.82	0.76	0.78	0.92	0.82
LRN	0.82	0.78	0.8	0.94	0.835
Res-soft	0.865	0.82	0.85	0.945	0.87
SNRSN	0.91	0.88	0.89	0.96	0.91

**Table 4:** Results of different networks.

Model	TPR	FPR
CNN	0.82	1.0
LRN	0.835	1.0
Res-soft	0.87	1.0
SNRSN	0.91	1.0
AFUCR-SNRSN	0.895	0.09

## Data Availability

Data sharing is not applicable to this article due to privacy.

## Abbreviations

The following abbreviations are used in this manuscript:

FANET	Flying ad-hoc network
UAVs	Unmanned aerial vehicles
SVM	Support vector machine
JNR	Jamming to noise ratio
DNNs	Deep neural networks
AC-VAEGAN	Auxiliary classifier variational auto-encoding generative adversarial network
1D-CNN	One-dimensional convolutional neural network
ResNet	Residual neural network
LRN	Lightweight residual network
HDC	Hybrid dilated convolution
STFT	Short-time Fourier transform
TF-LSTM	Time–frequency long short-term memory
Res-soft	Residual network based on soft fusion
SNCS	Simple non-local correction shrinkage
LIP	Local importance-based pooling
SNRSN	Simplified non-local residual shrinkage network
AFUCR	Acceptance factor-based unknown class recognition
DRSN	Deep residual shrinkage network
OCSVM	One-class vector machine
AM	Amplitude modulation
FM	Frequency modulation
TPR	True positive rate
FPR	False positive rate
TP	True positive
FN	False negative
FP	False positive
TN	True negative
